# Prevalence of staphylococcal toxin in food contaminated by *Staphylococcus* spp.: Protocol for a systematic review with meta-analysis

**DOI:** 10.1371/journal.pone.0282111

**Published:** 2023-02-21

**Authors:** Juliana Karla Garcia Ribeiro Freitas, Cristiane Fernandes de Assis, Thailla Raquel Moura de Oliveira, Cláudio Márcio de Medeiros Maia, Bruno Jonatan de Sousa, Gidyenne Christiane Bandeira Silva de Medeiros, Larissa Mont’Alverne Jucá Seabra, Karla Suzanne Florentino da Silva Chaves Damasceno

**Affiliations:** 1 Nutrition Postgraduate Program, Health Sciences Center, Federal University of Rio Grande do Norte (UFRN), Natal, RN, Brazil; 2 Department of Pharmacy, Health Sciences Center, Federal University of Rio Grande do Norte, Natal, RN, Brazil; 3 Department of Nutrition, Health Sciences Center, Federal University of Rio Grande do Norte (UFRN), Natal, RN, Brazil; 4 Systematic Review and Meta-Analysis Laboratory (Lab-SYS) CNPq-UFRN, Natal, RN, Brazil; Shahjalal University of Science and Technology, BANGLADESH

## Abstract

**Background:**

Food contamination by *Staphylococcus* spp. enterotoxigenic strains is quite common and despite underreporting caused by the short duration of clinical symptoms and lack of medical care, staphylococcal food poisoning is one of the most common Foodborne Diseases (FBD) in the world. This study describes a systematic review protocol with meta-analysis on the prevalence and types of staphylococcal enterotoxins in food, and the profile of contaminated foods.

**Methods:**

The research will be conducted through the selection of studies reporting the analysis of staphylococcal enterotoxins in food contaminated by *Staphylococcus* spp. Searches will happen on the following databases: Medline (OVID), GALE, Science Direct, CAB Direct (CABI), Google Scholar, in addition to manual search in the list of references of articles, directory of theses and dissertations, and countries’ health agencies. Reports will be imported into the application Rayyan. Two researchers will independently select studies and extract data, and a third reviewer will solve conflicting decisions. The primary outcome will be the identification of staphylococcal enterotoxins in food, and the secondary outcomes will include staphylococcal enterotoxin types and foods involved. To assess the risk of bias in the studies, the tool developed by the Joanna Briggs Institute (JBI) will be used. For data synthesis, a meta-analysis will be performed. However, in case that is not possible, a narrative synthesis of the most relevant results will be carried out.

**Discussion:**

This protocol will serve as the basis for a systematic review that aims to relate the results of existing studies on the staphylococcal enterotoxin prevalence and types in food, and the profile of the contaminated foods. The results will broaden the perception of food safety risks, highlight existing literature gaps, contribute to the study of the epidemiological profile and may guide the allocation of health resources for the development of preventive measures related.

**Systematic review registration:**

PROSPERO registration number: CRD42021258223.

## Introduction

Food quality is defined based on its different nutritional, sensory, health, and sanitary aspects [1]. In addition, more recent studies show the importance of adding environmental impact and sustainability, which involves the entire food system, to food quality criteria [2]. Foodborne contaminants are numerous; they include viruses and bacteria, fungi, parasites, chemicals, toxins, pesticides, heavy metals, and allergens that cause a range of pathological conditions [3, 4]. The presence or activity of microorganisms in food can affect its sensory quality, and when pathogenic microorganisms are present in a quantity above the allowed limit, food safety is compromised, which can have negative consequences for the health of the population [1].

Foodborne Diseases (FBD) have been considered one of the main public health problems worldwide, including in developed countries [5, 6]. Besides that, they are important causes of morbidity and mortality and therefore an important obstacle to global socioeconomic development [6, 7]. Investment in improving food safety management capacity may have significant economic and public health benefits [8].

A FBD is a syndrome generally consisting of gastrointestinal signs and symptoms, but extra-intestinal disorders can also occur in different organs, such as kidney, liver, central nervous system, among others [9]. The World Health Organization (WHO) has estimated that unsafe food has led to 600 million cases of FBD, 420,000 deaths, and the loss of 33 million years of healthy life worldwide [7].

Enterotoxins produced by pathogenic *Staphylococcus* strains are often involved in FBD outbreaks [10–12]. The main prerequisite for staphylococcal food poisoning is that the food is contaminated by an enterotoxigenic strain of *Staphylococcus* spp., which is considered a major global cause of FBD [13, 14]. Contamination can occur during food handling stages by carrying handlers [15–17] or through mastitis of dairy animals [5, 18–20].

In the genus *Staphylococcus* spp., there are 53 species and 28 subspecies [6, 21], some frequently involved in FBD outbreaks due to the production of several types of enterotoxins [5, 10, 12]. The species have several aspects of virulence, which are coded through their different classes mobile genetic elements that determine the production of hemolysins, coagulases, lipases and mainly enterotoxins, which explains the different types of enterotoxins produced, the characteristics of virulence and resistance [22, 23].

It is important to highlight that some studies show the existence of strains resistant to antibiotics [14, 17, 21, 24–26] and disinfectants [13], and that many strains are capable of producing 2 or 3 types of enterotoxins simultaneously [27], which is worrying since they make it difficult to investigate outbreaks, prevent and treat diseases caused by *Staphylococcus* spp. [17], more importantly because resistance can be genetically transferred among the various *Staphylococcus* species, leading to an increase and spread of resistant enterotoxigenic staphylococci and/or pathogenic staphylococci [13].

In order for enterotoxins to be produced, there must be suitable conditions of temperature, pH and water activity [28]. After being produced by the strains, staphylococcal enterotoxins (SE) are resistant to various environmental situations, such as freezing, drying, heat treatment and low pH–conditions which easily destroy the enterotoxin-producing strains themselves [5].

According to the Food and Drug Administration (FDA) the intoxication dose of SE is less than 1.0 μg, which in the case of *S*. *aureus*, is reached when its population exceeds 100,000 organisms/g in the food [28]. However, in more sensitive people, the ingestion of 100 to 200 ng of enterotoxin can already cause symptoms [28, 29]. In 2003, Asao *et al*. [30] suggested that the large outbreak in Osaka in 2000 was caused by small amounts (20-100ng) of staphylococcal enterotoxin A (SEA) in reconstituted milk, however, 2 years later a study published by Ikeda *et al*. [31] clearly indicated the presence of staphylococcal enterotoxin H (SEH) in the food involved in the outbreak, which proves that the food poisoning was caused by multiple toxins.

Staphylococcal poisoning usually has a fast symptom onset (1 to 7 hours) and often acute, depending on the individual’s susceptibility to the toxin, the amount of toxin ingested, and the individual’s general health [28]. Symptoms commonly include nausea, abdominal pain, vomiting, and diarrhea. In more severe cases, in vulnerable groups, major complications may occur [14, 28].

So far, worldwide prevalence records of staphylococcal enterotoxin in food contaminated by *Staphylococcus* spp. are not available. Several studies on the topic, including reports of possible outbreaks involving this bacterium, do not have conclusive results, since research on the bacterium [32–35] or on the gene responsible for the production of the SE is observed, which do not necessarily confirm the presence of the toxin in food [6, 12, 27, 35–37]. Therefore, awareness and discussion of the occurrence of SE in food is important to put this topic in evidence and highlight the need to identify the toxin effectively and quickly, aiming at food safety.

Given the relevance of this topic, this study protocol seeks to describe the procedures for a systematic review on the worldwide prevalence of staphylococcal enterotoxin in foods contaminated by *Staphylococcus* spp.

## Methods

### Study registration

This systematic review protocol was structured according to the guidelines provided in the PRISMA-P (Preferred Reporting Items for Systematic Review and Meta-Analyses Protocols) [38] (S1 File), and registered in the PROSPERO database (International Prospective Register of Systematic Reviews), with registration number CRD42021258223. In such protocol are described the eligibility criteria, databases selected for searches, evaluated variables, and how heterogeneity will be treated, allowing this systematic review to be impartial, transparent and reproducible [39].

### Eligibility criteria

The eligibility criteria (Table 1) for the study were defined based on the PECOS classification (Population, Exposure, Comparator, Outcomes and Study Types)–a tool to guide the research and define search strategies [39].

**Table 1:** Eligibility criteria based on PECOS (Population, Exposure, Comparator, Outcomes and Study Types).

PECOS	Inclusion	Exclusion
Population	• Primary studies reporting staphylococcal enterotoxin (SE) analysis in food.	• Studies on SE in humans, domestic or food-producing animals, fomites or surfaces. • Studies only aimed at the microorganism or gene responsible for the production of the SE.
Exposure	• Foods exposed to *Staphylococcus* spp.	• Studies which are **not** connected to the exposition of food to *Staphylococcus* spp. • Studies which report direct contamination of food with SE, in laboratory settings.
Comparator	• Not applicable.	• Not applicable.
Outcomes	Primary • Quantification of SE in foods contaminated with *Staphylococcus* spp; Secondary • Types of SE found in food; Types of food contaminated with *Staphylococcus* spp.	Limited to the presence of *Staphylococcus* spp; • Limited to the presence of the gene responsible for the production of the SE.
Study types	• Primary cross-sectional studies.	-

### Search strategy

The search strategies (Table 2) were designed considering the topic and the particularities of the information desired to answer the research question: What is the worldwide prevalence of staphylococcal enterotoxins in food contaminated by *Staphylococcus* spp.?

**Table 2:** Search strategy applied on Medline (OVID).

Database	Search strategy
Medline (OVID)	Line 1: staphylococcal enterotoxin*.ti,ab.
	Line 2: contaminated food*.ti,ab.
	Line 3: food contamination.ti,ab.
	Line 4: staphylococcal food poisoning.ti,ab.
	Line 5: disease* outbreak.ti,ab.
	Line 6: foodborne disease*.ti,ab.
	Line 7: food poisoning.ti,ab.
	Line 8: Staphylococc*.ti,ab.
	Line 9: outbreak.ti,ab.
	Line 10: 2 or 3 or 4 or 5 or 6 or 7 or 8 or 9
	Line 11: 1 and 10

The terms used were defined based on information from PECOS, which would convey the criteria in formulating a search strategy. Terms related to population (staphylococcal enterotoxins) and exposure (contaminated food, food contamination, staphylococcal food poisoning, disease outbreak, foodborne disease, food poisoning, *Staphylococcus* spp.) will be used. The operators ‘AND’, ‘OR’ and ‘AND NOT’ will also be used in the construction of the search strategy which will be adapted to meet the specifications of the search syntax of each database.

The following databases will be used: Medline (OVID), GALE, Science Direct, CAB Direct (CABI), and Google Scholar. In addition, manual search will be done in records’ reference lists, directory of theses and dissertations, and countries’ health agencies. Gray literature will be included. Studies from the last 20 years (2002–2022) will be included. There will be no language restriction.

### Screening procedure

After running the search strategies, all records found will be imported into *Rayyan Intelligent Systematic Review®* [40]. The selection of studies will be done without interference or contact between researchers, so that influences in the decision process are avoided.

Initially, automatic duplicate detection will be performed, and then Researcher A will manually decide to exclude potentially duplicate studies. After that, Researchers A and B will independently read the titles and abstracts to screen the studies which meet the eligibility criteria for inclusion and exclusion. In case there are conflicting decisions, Researcher C will decide on the inclusion or exclusion of the study. After this step, the selected articles will undergo full reading by Researchers A and B independently, so that eligibility is confirmed. Conflicting decisions will be resolved by consensus and, when necessary, by Researcher C.

### Data extraction

Data extraction will be performed by filling out an electronic spreadsheet with a detailed description of the main information in the selected studies. This step will also be done by two researchers independently, in order to avoid measurement bias, which occurs due to misinterpretation or even the loss of important data to be collected. If necessary, records’ authors may be contacted to clarify possible doubts. Data mismatching will also be resolved by consensus and, when necessary, by a third researcher.

In order for this information to be compared in a clear and objective way, simplifying the verification process, it was previously established which data should be collected, such as: study title, author, year of publication, journal in which the study was published, objective of the study. Besides that, data will be collected on the methodology used for the analysis of the toxin, and analyses results, including food or food group analyzed, quantity of analyzed foods (number of samples), in which and how many of the analyzed foods the toxin was detected, prevalence (%), types of SE found and SE concentration (ng or equivalent unit).

If the studies present the following pieces of information, they will also be collected: confidence interval (CI), significance level adopted, detection limit of the method used, minimum dose for intoxication.

### Risk of bias assessment

After the selection of the primary studies that will be included in this systematic review, critical assessment of their quality will be carried out, having as reference the tool developed by the Joana Briggs Institute [41] specifically for systematic reviews of prevalence and incidence studies. Questions related to the study sample (representativeness and size), validity of the methodology used and standardization, description of the study design, result analysis and statistical analysis of data will be evaluated. The tool was interpreted for better application to the study (S2 File) and each item must be marked: “yes”, “no”, “unclear”, or “not applicable”. Questions 3 and 9 were considered not applicable to this research. Question 3, which refers to the sample size, was excluded considering that a positive result for enterotoxin from only 1 food sample would be enough to evaluate the study. Question 9 assesses the response rate and is not applicable due to this study’s population (food).

Seven questions will be considered, so the maximum score will be 7. The methodological quality of the studies will be categorized as low risk of bias (> 5 answers “yes”), moderate risk of bias (4–5 answers “yes”), and high risk of bias (< 4 answers “yes”). There will be a narrative summary of the overall methodological quality of the studies included, which will be supported by a table with the results of the critical evaluation and classification adopted in this research, which will clarify the accuracy of the evaluation of the studies included and clarify the reasons for including deficient studies.

### Data synthesis

The main outcome evaluated will be the prevalence of staphylococcal enterotoxin in foods analyzed and contaminated by *Staphylococcus* spp. Such outcome will be calculated considering the number of foods contaminated with some type of SE among foods analyzed and contaminated by *Staphylococcus* spp. Secondary outcomes such as types of SE found in contaminated foods and types of foods will also be analyzed.

The heterogeneity of the results will be evaluated using I-square (I^2^) and chi-square (χ^2^) statistics. The χ^2^ test is one of the most used to assess the level of significance of heterogeneity, if there is little variation between studies, with a significance level of p < 0.1 [42, 43] and when values of the magnitude of heterogeneity, evaluated by the I^2^, are less than 50%, it is possible to conclude that the synthesis of results through meta-analysis is feasible [42–44]. For the meta-analysis result, data will be presented estimating a 95% confidence level (95% CI) for the corresponding effect size.

Critical examination of systematic reviews for publication bias should be considered a routine procedure. If possible, this item will be evaluated using funnel plots–a simple analysis of this chart will be a useful test for the probable presence of bias in meta-analyses. The statistical tests of Egger [45] and Begg [46] will be performed in a complementary way and publication bias will be considered when p < 0.10.

Due to such a broad and variable population group (samples of various types of food), it is expected that the results will have a greater heterogeneity. To avoid such effect, if necessary, food subgroups with similar characteristics will be formed.

Random variations between the point estimates of the primary study subgroup and the possible source of heterogeneity analysis can be made considering the types of SE found, food groups with similar characteristics, toxin detection techniques, and countries.

If it is not possible to quantitatively synthesize the data through meta-analysis, the most relevant results will be summarized, described and discussed, considering the risk of bias and seeking to provide conclusions on the evidence.

### Quality of evidence appraisal

GRADE (Grading of Recommendations Assessment, Development and Evaluation) will be used for assessing the quality of evidence [47].

## Discussion

Food contamination by *Staphylococcus* spp. strains which produce enterotoxins is quite common due to the high prevalence of human carriers [15–17] and raw material contaminated at source, mainly milk [5, 18–20].

Staphylococcal food poisoning is one of the most common FBD in the world [13, 14], however, established investigation protocols are generally not followed and medical, epidemiological and laboratory data are often underreported. Clinically, the disease caused by these toxins usually lasts a short period, and therefore patients ignore medical attention, or doctors do not request laboratory tests, making it difficult to identify the etiological agents of outbreaks [5, 6]. Besides that, SE analyses in food are not simple and affordable, thus they are not commonly conducted by laboratories [5], being laboratorial analyses frequently limited to identifying *Staphylococcus* sp. strains. These aspects are undoubtedly this research’s main limiting factors.

Besides that, other limiting factors will be the difficulty of accessing studies in progress and records not registered in the selected databases. Methodological limitations of primary studies and reporting bias will have their quality assessed by the tool used in this study [41].

The integration of the results of studies on the occurrence of SE, the knowledge related to the profile of the foods most often involved, and the types of SE found is a step towards expanding the general perception of risks to food safety, highlighting the existing gaps on the topic, contributing to the study of the epidemiological profile, and facilitating the adoption of more effective prevention measures and policies, which will contribute to the improvement of food quality [48, 49].

This study protocol systematically describes the methodological procedures planned to be used in a systematic review on the worldwide prevalence of staphylococcal enterotoxin in foods contaminated by *Staphylococcus* spp. The expectation is that it will help to conduct the research in a way that guarantees reliability, transparency, and reproducibility. Ultimately, the results of this work may guide the allocation of health resources for the development of preventive measures related to Good Manufacturing Practices (GMP) in Food.

## Supporting information

S1 File PRISMA-P 2015 checklist. (DOCX)

S2 File Risk of bias evaluation. (PDF)
