# Supplementary material for: Prevalence of staphylococcal toxin in food contaminated by Staphylococcus spp.: Protocol for a systematic review with meta-analysis
Source: PLoS One. 2023 Feb 21;18(2):e0282111. doi: 10.1371/journal.pone.0282111 (PMC9942949; doi:10.1371/journal.pone.0282111)
Supplement: S2 File — (PDF) [file pone.0282111.s002.pdf]

S2 File. Risk of bias evaluation.

<u>Article: Prevalence of staphylococcal toxin in food contaminated by *Staphylococcus* spp.: protocol for a systematic review with meta-analysis</u>

JBI CRITICAL APPRAISAL CHECKLIST FOR STUDIES REPORTING PREVALENCE DATA*

Reviewer: Date:

Author: Year: Record Number:

	YES	NO	UC	NA
1. Was the sample frame appropriate to address the target population?	☐	☐	☐	☐
2. Were study participants sampled in an appropriate way?	☐	☐	☐	☐
3. Was the sample size adequate?	☐	☐	☐	X
4. Were the study subjects and the setting described in detail?	☐	☐	☐	☐
5. Was the data analysis conducted with sufficient coverage of the identified sample?	☐	☐	☐	☐
6. Were valid methods used for the identification of the condition?	☐	☐	☐	☐
7. Was the condition measured in a standard, reliable way for all participants?	☐	☐	☐	☐
8. Was there appropriate statistical analysis?	☐	☐	☐	☐
9. Was the response rate adequate, and if not, was the low response rate managed appropriately?	☐	☐	☐	X

UC: Unclear NA: Not Applicable

Risk of bias classification – Sum of answers 'YES':
[ ] > 5 answers 'YES'. Study presents **low** risk of bias.
[ ] 4-5 answers 'YES'. Study presents **medium** risk of bias.
[ ] < 4 answers 'YES'. Study presents **high** risk of bias.

Attempt contact with authors for more information? [ ] YES [ ] NO

Comment(s):

---

*Munn Z, Moola S, Lisy K, Riitano D, Tufanaru C. (2015) Methodological guidance for systematic reviews of observational epidemiological studies reporting prevalence and incidence data. **Int J Evid Based Healthc.** 2015; 13:147–153.

#	ORIGINAL QUESTION	EXPLANATION OF PREVALENCE CRITICAL APPRAISAL	HOW DOES IT SPECIFICALLY APPLY TO OUR STUDY? / KEY INTERPRETATION
1.	Was the sample frame appropriate to address the target population?	This question relies upon knowledge of the broader characteristics of the population of interest and the geographical area. If the study is of women with breast cancer, knowledge of at least the characteristics, demographics and medical history is needed.<br><br>The term "target population" should not be taken to infer every individual from everywhere or with similar disease or exposure characteristics. Instead, give consideration to specific population characteristics in the study, including age range, gender, morbidities, medications, and other potentially influential factors.<br><br>For example, a sample frame may not be appropriate to address the target population if a certain group has been used (such as those working for one organization, or one profession) and the results then inferred to the target population (i.e. working adults).<br><br>A sample frame may be appropriate when it includes almost all the members of the target population (i.e. a census, or a complete list of participants or complete registry data).	**Was the sample used appropriate for the purpose of the study, considering the type of food and the location where it was collected?**<br><br>- Nature of the samples (Type of food, was the sample part of suspectedly contaminated food?)<br><br>- City, country of origin, and collection site<br><br>**YES:** When the following is specified:<br>• Type of food;<br>• Reason for analyzing the food (e.g., potential outbreak)<br>• Collection site (city, country)<br><br>**NO:** When the collected sample is not a suspectedly contaminated food or no adequate reason for its inclusion is described in the study (e.g., the food linked to the outbreak is not recovered and other food is analyzed).<br><br>**UNCLEAR:** When any aspect is unclear or when information is not provided.
2.	Were study participants sampled in an appropriate way?	Studies may report random sampling from a population, and the methods section should report how sampling was performed. Random probabilistic sampling from a defined subset of the population (sample frame) should be employed in most cases, however, random probabilistic sampling is not needed when everyone in the sampling frame will be included/analysed.<br><br>For example, reporting on all the data from a good census is appropriate as a good census will identify everybody. When using cluster sampling, such as a random sample of villages within a region, the methods need to be clearly stated as the precision of the final prevalence estimate incorporates the clustering effect. Convenience samples, such as a street survey or interviewing lots of people at public gatherings are not considered to provide a representative sample of the base population.	**Were the sampling process and collection method described? Was the study (results / discussion / conclusion) conducted in a coherent way?**<br><br>Expected description:<br>• Transport at refrigeration temperature and using isothermal boxes<br>• No longer than 36 hours from collection to analysis<br><br>**YES:** Information available and adequate.<br><br>**NO:** Information available and <u>inadequate</u>.<br><br>**UNCLEAR:** Information unavailable or inappropriately described, leading to doubts.
3.	Was the sample size adequate?	The larger the sample, the narrower will be the confidence interval around the prevalence estimate, making the results more precise.<br><br>An adequate sample size is important to ensure good precision of the final estimate. Ideally we are looking for evidence that the authors conducted a sample size calculation to determine an	**NOT APPLICABLE.**<br><br>Question excluded since a positive result for enterotoxin from at least one food sample would be enough to evaluate the study.

		adequate sample size. This will estimate how many subjects are needed to produce a reliable estimate of the measure(s) of interest.<br><br>For conditions with a low prevalence, a larger sample size is needed. Also consider sample sizes for subgroup (or characteristics) analyses, and whether these are appropriate. Sometimes, the study will be large enough (as in large national surveys) whereby a sample size calculation is not required. In these cases, sample size can be considered adequate.<br><br>When there is no sample size calculation and it is not a large national survey, the reviewers may consider conducting their own sample size analysis using the following formula:<br>(Naing et al. 2006, Daniel 1999)<br><br>n = Z2P(1-P) d2<br><br>Where:<br>n= sample size<br>Z = Z statistic for a level of confidence<br>P = Expected prevalence or proportion (in proportion of one; if 20%, P = 0.2)<br>d = precision (in proportion of one; if 5%, d=0.05)<br><br>Ref:<br>Naing L, Winn T, Rusli BN. Practical issues in calculating the sample size for prevalence studies Archives of Orofacial Sciences. 2006;1:9-14.<br><br>Daniel WW. Biostatistics: A Foundation for Analysis in the Health Sciences. Edition. 7th ed. New York: John Wiley & Sons. 1999.	
4.	Were the study subjects and the setting described in detail?	Certain diseases or conditions vary in prevalence across different geographic regions and populations (e. g. Women vs. Men, sociodemographic variables between countries).<br><br>The study sample should be described in sufficient detail so that other researchers can determine if it is comparable to the population of interest to them.	**Are the different ways of samples treatment described according to the type of food?**<br><br>**YES:** There were no differences in sample treatment. OR<br>Differences in methodology were described according to the type of food.<br><br>**NO:** Samples were not treated properly.<br><br>**UNCLEAR:** The authors did not describe how the different samples were treated. Relevant details were not available.

5.	Was the data analysis conducted with sufficient coverage of the identified sample?	Coverage bias can occur when not all subgroups of the identified sample respond at the same rate.<br><br>For instance, you may have a very high response rate overall for your study, but the response rate for a certain subgroup (i.e. older adults) may be quite low.	**If there are different subgroups of foods, was a similar number of samples analyzed in all of them?**<br><br>**YES:**<br>• There is no difference in the number of samples between subgroups/types and between methodology and results<br>• The reasons are described for differences, if any, in the number of samples identified and analyzed or differences in sample number between subgroups/types of food<br><br>**NO:** When a difference is noticed in the number of samples mentioned in the methodology and in the result section and reasons for so are not listed<br><br>**UNCLEAR:** Information unavailable or inappropriately described, leading to doubts.
6.	Were valid methods used for the identification of the condition?	Here we are looking for measurement or classification bias.<br><br>Many health problems are not easily diagnosed or defined and some measures may not be capable of including or excluding appropriate levels or stages of the health problem.<br><br>If the outcomes were assessed based on existing definitions or diagnostic criteria, then the answer to this question is likely to be yes.<br><br>If the outcomes were assessed using observer reported, or self-reported scales, the risk of over- or under-reporting is increased, and objectivity is compromised.<br><br>Importantly, determine if the measurement tools used were validated instruments as this has a significant impact on outcome assessment validity	**Were valid methods used for toxin determination?**<br><br>Examples of valid methods:<br>• Ouchterlony and variations<br>• ELIZA (Enzyme-Linked Immunosorbent Assay) kits<br>• Radioimmunoassay (RIA)<br>• Western blot<br>• Latex agglutination/fixation test<br>• C-Reactive Protein (CRP) Test<br>• Microarray<br><br>**YES:** When described and valid.<br><br>**NO:**<br>• When described and <u>not</u> valid.<br>• Bioassays<br><br>**UNCLEAR:** When the method is not mentioned.
7.	Was the condition measured in a standard, reliable way for all participants?	Considerable judgment is required to determine the presence of some health outcomes.<br><br>Having established the validity of the outcome measurement instrument (see item 6 of this scale), it is important to establish how the measurement was conducted.	**Was the condition measured in the same way for all samples or was there justification for adaptations made?**<br><br>**YES:** When the following was described and adequate:<br>• sample collection.<br>• sample treatment.<br>• preparation of standard solutions.<br>• equipment used and whether it is calibrated.

		Were those involved in collecting data trained or educated in the use of the instrument/s? If there was more than one data collector, were they similar in terms of level of education, clinical or research experience, or level of responsibility in the piece of research being appraised?<br><br>When there was more than one observer or collector, was there comparison of results from across the observers? Was the condition measured in the same way for all participants?	• calculations to determine the results.<br><br>- Were the limits of detection (LD) and limits of quantification (LQ) the same in the analysis of different samples of the same food?<br><br>**NO:** When information is available, but inadequate.<br><br>**UNCLEAR:** Information unavailable.
8.	Was there appropriate statistical analysis?	Importantly, the numerator and denominator should be clearly reported, and percentages should be given with confidence intervals.<br><br>The methods section should be detailed enough for reviewers to identify the analytical technique used and how specific variables were measured.<br><br>Additionally, it is also important to assess the appropriateness of the analytical strategy in terms of the assumptions associated with the approach as differing methods of analysis are based on differing assumptions about the data and how it will respond.	**Was any statistical method used? Was there a description of it?**<br><br>**YES:** An appropriate statistical methos was used for analysis of the results.<br><br>**NO:** An appropriate statistical methos was not used.<br><br>**UNCLEAR:** Information unavailable.
9.	Was the response rate adequate, and if not, was the low response rate managed appropriately?	A large number of dropouts, refusals or “not founds” amongst selected subjects may diminish a study’s validity, as can a low response rates for survey studies.<br><br>The authors should clearly discuss the response rate and any reasons for non-response and compare persons in the study to those not in the study, particularly with regards to their socio-demographic characteristics.<br><br>If reasons for non-response appear to be unrelated to the outcome measured and the characteristics of non-responders are comparable to those who do respond in the study (addressed in question 5, coverage bias), the researchers may be able to justify a more modest response rate.	**NOT APPLICABLE.**<br><br>Since the population of this study (food) does not relate to response rate, this item was considered not applicable.
